# A phase 1 study of intra-arterial CBL0137 in extremity melanomas and sarcomas

**DOI:** 10.1016/j.suronc.2025.102243

**Published:** 2025-06-07

**Authors:** Joseph J. Skitzki, Minhyung Kim, Daniel T. Fisher, John M. Kane, Han Yu, Kayla Catalfamo, Garin Tomaszewski, Michael Petroziello, Andrei Purmal, Katerina V. Gurova, Andrei V. Gudkov

**Affiliations:** a Department of General Surgery, Cleveland Clinic, Cleveland, OH, USA; b Department of Immunology, Roswell Park Comprehensive Cancer Center, Buffalo, NY, USA; c Department of Comparative Oncology, Roswell Park Comprehensive Cancer Center, Buffalo, NY, USA; d Department of Surgical Oncology, Roswell Park Comprehensive Cancer Center, Buffalo, NY, USA; e Department of Biostatistics and Bioinformatics, Roswell Park Comprehensive Cancer Center, Buffalo, NY, USA; f Department of Radiology/Interventional Radiology, Roswell Park Comprehensive Cancer Center, Buffalo, NY, USA; g Incuron, Inc., Buffalo, NY, USA; h Department of Cell Stress Biology, Roswell Park Comprehensive Cancer Center, Buffalo, NY, USA

**Keywords:** Melanoma, Sarcoma, Regional therapy, ILI, ILP, CBL0137

## Abstract

**Introduction:**

Regional therapies for cancer leverage the ability to isolate the circulation to a diseased extremity or organ and deliver high doses of chemotherapy that would be systemically prohibitive due to toxicity. Virtually all regional therapies utilize the original chemotherapy agent, melphalan, which requires circulatory isolation. CBL0137 is a small molecule with multiple anti-tumor effects when given intra-arterially (IA) that shows similar efficacy to melphalan in preclinical models, but without the need for circulatory isolation.

**Materials and methods:**

Patients with advanced, unresectable melanoma or sarcoma (n = 5, sarcoma 60 %, melanoma 40 %) of the extremity entered a rapid dose-escalation phase of a clinical trial of IA CBL0137. CBL0137 was administered via a single IA catheter placed proximal to the site of tumor(s) in the affected extremity and delivered over 15 min. Primary objective was to define dose-limiting toxicities with secondary objectives of assessing response and pharmacokinetics (PK).

**Results:**

The treatments were well tolerated with minimal to no toxicity for all patients. PK data showed predictable, dose-dependent drug exposures with rapid tissue uptake and markedly decreased systemic concentrations as compared to matched intravenous dosing data. CBL0137 preferentially accumulated within tumor tissue as compared to surrounding normal tissue in the infused limb without the need for tourniquet. Sixty percent of patients treated in this protocol would not have been eligible for standard regional therapies with one patient demonstrating prolonged disease stability while avoiding major amputation.

**Conclusions:**

The historic restrictions of standard regional therapies may be overcome with IA CBL0137, and this treatment is potentially applicable to a wide range of cancers beyond the extremities.

## 1. Introduction

Regional therapies for cancer, including isolated limb perfusion (ILP) and isolated limb infusion (ILI), take advantage of the ability to isolate the circulation to a diseased extremity and deliver high doses of chemotherapy that would be prohibitively toxic if given systemically [1]. Reduced systemic toxicity is dependent upon chemotherapy being circulated via arterial and venous access that is excluded from the systemic circulation via a tourniquet. After treatment completion, the circulated chemotherapy is drained and the limb flushed so that systemic chemotherapy exposure is eliminated when the tourniquet is released [2]. Historically, ILP and ILI were used to treat extremity in-transit cutaneous malignancies including melanoma or locally advanced sarcomas as a potentially curative and/or palliative alternative to amputation [3,4]. High volume experience has yielded established treatment paradigms for both ILP and ILI with a clear benefit based upon their disparate properties [5,6]. The first ILP was performed in 1957 by Creech et al. and involved the administration of phenylalanine mustard (PAM), now commonly known as melphalan [7]. In 1997, Thompson et al. developed ILI by substituting smaller diameter catheters placed percutaneously under radiographic guidance [8]. While ILI is technically easier to perform, the dependence upon melphalan and the need to isolate the circulation to achieve therapeutic doses and minimize systemic toxicity remains. Furthermore, local toxicity in the form compartment syndrome or tissue loss requiring amputation may be devastating [9]. Recent advances in immunotherapy, small molecule inhibitors, and injectable therapies have relegated ILP and ILI procedures to limited clinical use [10,11]. As a one-time, low-cost treatment with complete response rates of 57–76 % and 27–38 % for ILP and ILI, respectively [12], regional therapies for cancer remain an intriguing concept with renewed interests in combinatorial strategies [13]. However, the inherent properties of ILP and ILI including technical complexity, patient selection, and the continued chemotherapy backbone of melphalan are significantly limiting. A paradigm shift from long-standing historical concepts is required to advance regional therapies for cancer.

Evaluation of novel agents for regional therapy applications was only recently made possible via the development of a clinically relevant immunocompetent mouse model [14]. The novel agent CBL0137 was shown in murine models to exhibit properties that favored applications to a regional approach, specifically demonstrating preferential tumor uptake compared to normal tissue when given intra-arterially and anti-tumor activity at doses that were a fraction of the intravenous MTD; thus, eliminating the need for tourniquet and venous catheterization [15]. The intra-arterial (IA) focused delivery of CBL0137 was necessary for anti-tumor effects that did not occur with systemic intravenous (IV) delivery at even higher doses in these preclinical models. Directly comparing IA vs. IV delivery at the same doses in this mouse melanoma model showed an at least 8-fold increase in CBL0137 concentration within tumor tissue [15]. Modest results from a clinical trial of intravenous CBL0137 where the intravenous route was minimally efficacious [16] would further suggest that an IA approach could be utilized clinically to potentiate anti-tumor responses. IA CBL0137 showed anti-tumor activity, but less toxicity than melphalan in murine preclinical models due to several unique characteristics.

CBL0137 is a water-soluble, highly cell-permeable carbazole-based small molecule that intercalates into DNA without causing DNA damage. Following tumor cell exposure to CBL0137, the FACT (FAcilitates Chromatic Transcription) chromatin remodeling complex becomes tightly associated with chromatin causing depletion of functional FACT, leading to activation of the p53 tumor suppressor (via phosphorylation by FACT-associated CK2) and inhibition of pathways dependent upon FACT [17] including NF-κB and heat shock factor 1 (HSF1) [18]. Tumor cells may be preferentially susceptible to CBL0137 while normal differentiated cells are resistant due to the absence of FACT expression, a reduced reliance upon FACT-dependent pathways, and less chromatin accessibility as compared to tumor cells [19].

**Fig. 1. –:**
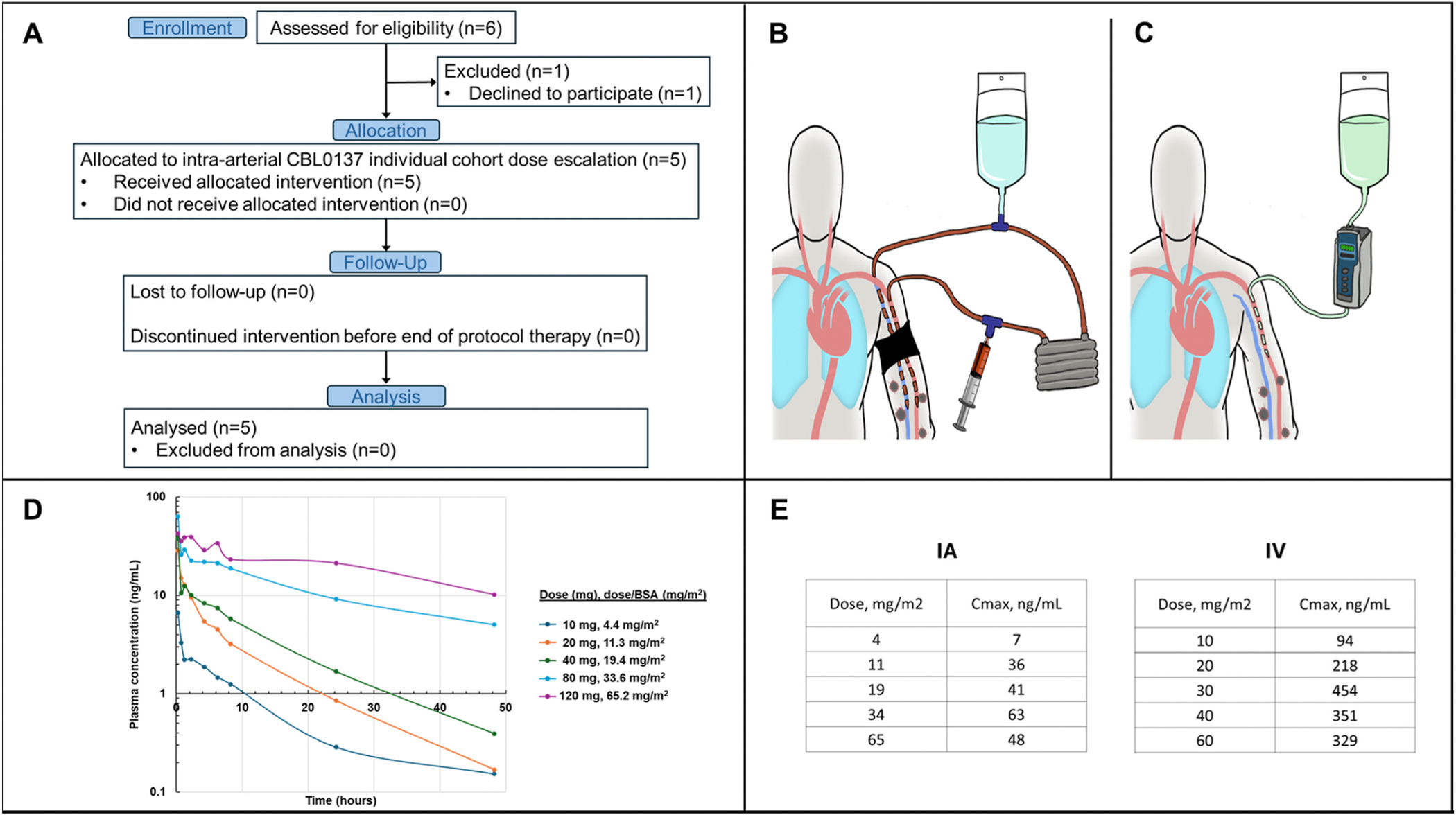
Simplified IA infusion produces consistent dose-dependent PK. A) CONSORT diagram of clinical trial. **B**) During ILI, the circulation to the extremity is isolated with a tourniquet and controlled with arterial and venous catheters for circulation of melphalan. **C**) A single intra-arterial catheter is placed and CBL0137 is infused over 15 min without the need for circulatory isolation. **D**) Increasing doses of CBL0137 were directly related to higher initial plasma values and slower clearance over time. **E**) Maximum systemic concentration in plasma was approximately a log-fold lower for IA delivery as compared to IV delivery confirming that circulatory isolation was not necessary.

Given the compelling preclinical and clinical data, a Phase I rapid dose escalation study was performed to assess the primary objective of toxicity, and secondary objectives of response, pharmacokinetics, and tissue uptake of CBL0137 (Fig. 1A). IA infusion was simplified from standard ILI (Fig. 1B) by having only a single intra-arterial catheter placed proximal to the tumor sites, no tourniquet or isolation of the circulation, no intravenous catheter placement, and a brief continuous 15-min infusion of the drug via a standard pump (Fig. 1C). Herein we report the results of the Acceleration Stage and first patient of the Escalation Stage that showed clinical feasibility and favorable pharmacokinetics of IA administration of CBL0137 that was well-tolerated and accumulated in tumor tissue preferentially. A total of five patients have been enrolled in this study and received intervention while one patient dropped out before the intervention. Two patients have subsequently received repeat dosing as part of a standard dose escalation phase that seeks the MTD for IA infusion.

## 2. Patients and methods

### 2.1. Patients

Patients with advanced extremity melanoma or sarcoma were evaluated for entry into an Institute Review Board approved clinical trial (ClinicalTrials.gov identifier: NCT03727789) at Roswell Park Comprehensive Cancer Center, Buffalo, NY. Major inclusion criteria included age >18 years, anticipated life expectancy >6 months, ECOG ≤2, ± prior limb directed therapy ILI or ILP, histologically proven and treatment refractory melanoma or sarcoma not amenable to surgical resection without major amputation, stable distant metastases (for ≥2 months if present), and a palpable pulse in the affected extremity. A notable exclusion criterion unique to CBL0137 was QT prolongation with mean QTcF values of >450 msec and females with QTcF values of >470 msec, patients who were known to have congenital prolonged QT syndromes, or patients actively taking medications known to cause prolonged QT intervals on ECG. A complete list of the inclusion and exclusion criteria can be found at https://clinicaltrials.gov/study/NCT03727789. Eligible patients who gave informed consent were enrolled in the protocol. Relevant demographic, tumor-specific, and pharmacokinetic (PK)/tissue concentration data were recorded.

### 2.2. Investigational drug

GMP-grade CBL0137 was provided gratis by Incuron, Inc. (Buffalo, NY, 14203). Roswell Park Comprehensive Cancer Center sponsored the FDA IND for this clinical trial.

### 2.3. Dose selection and escalation

In murine models, the optimally efficacious and safe intra-arterial dose (0.5 mg/25 g mouse; 20 mg/kg) was 1/3 of the intra-arterial MTD (1.5 mg/25 g mouse; 60 mg/kg) and 1/4 of the systemic MTD (2.0 mg/25 g mouse; 80 mg/kg) [15]. Based on allometric scaling as described in FDA guidance [20], a mouse dose of 20 mg/kg corresponds to a human dose of 1.6 mg/kg translating to a Human Equivalent Dose (HEDmouse) of ~110 mg. A human dose of 110 mg is < 1/4 of the dose of 320 mg/m^2^ (absolute dose range of 512–608 mg) that was well tolerated systemically in a trial evaluating IV administration of CBL0137 in patients with solid tumors [16]. As the primary toxicity associated with intra-arterial administration in the mouse was local toxicity (muscle injury or vascular damage), a substantial safety factor (≥10) was necessary in selecting the human starting dose. Thus, the clinical intra-arterial CBL0137 starting dose level was set at 10 mg. As absolute dose appeared to correlate with both efficacy and safety preclinically, CBL0137 was given at a fixed dose (mg/subject) without correction for limb volume, lymphedema, or body mass.

**Fig. 2. –:**
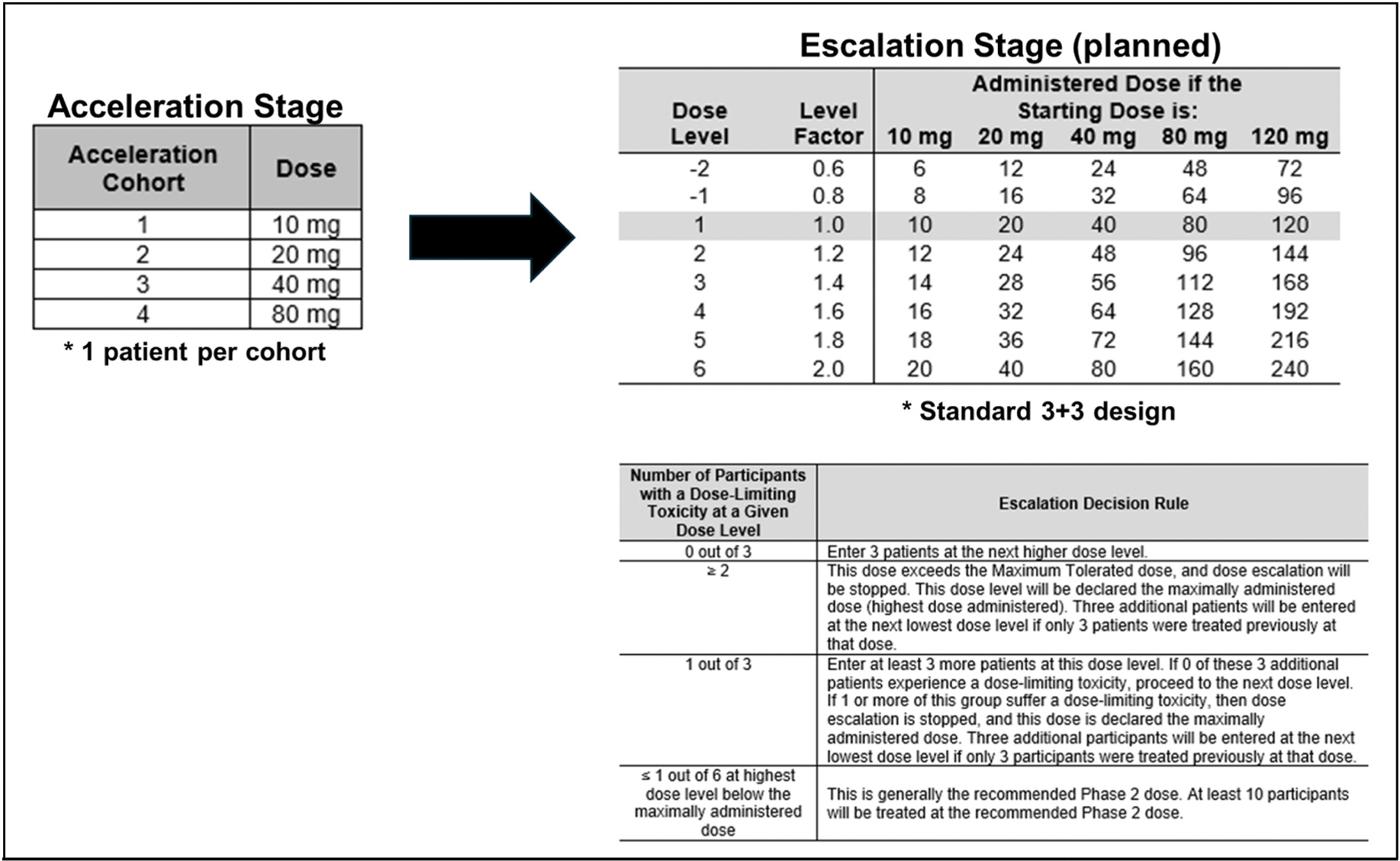
Dosing Schema consisted of a single patient cohort Acceleration Stage followed by an Escalation Stage. Rules for Escalation Stage are defined by standard 3 + 3 design. Results are reported from the Acceleration Stage and the first patient of the Escalation Stage.

As the DLT for intra-arterial CBL0137 used for melanoma or sarcoma of the extremities in humans is unknown, dose-escalation of CBL0137 is incorporated in this study design. Given that a substantial safety factor is used in calculating the starting dose, an initial single-subject dose-escalation paradigm is proposed to rapidly advance the dose and minimize the number of subjects who are administered potentially ineffective doses. An Acceleration Stage starting dose that allowed up to 4 dose doublings below the lower end of the projected MTD range was selected based on similar protocols [21]. The anticipated lower end of the MTD was 100 mg and the following Acceleration Dose schedule was applied with single patient dose cohorts: Cohort 1–10 mg, Cohort 2–20 mg, Cohort 3–40 mg, and Cohort 4–80 mg. A single patient in Cohort 5 received 120 mg as the starting dose for a planned future Escalation Stage (3 + 3 Design) and was included in this report (Fig. 2).

### 2.4. Treatment plan

All patients were treated in the interventional radiology suite and admitted for 48–72 h following treatment. Patients were npo after midnight and received conscious sedation while being continuously monitored. Patients underwent placement of a 5F intra-arterial catheter (Cook Medical, Bloomington, IN) via ultrasound guidance. The tumor location and patient anatomic variables and characteristics determined the placement of the catheter either ipsilateral, contralateral, antegrade, or retrograde. The correct position of the catheter tip was confirmed upstream of the tumor(s) radiographically. CBL0137 was diluted with sterile D5 W to a total volume of 250 ml and infused via an Alaris^™^ infusion pump (BD, Franklin Lakes, NJ) completely over 15 min. At the end of the infusion, the catheter was removed and direct pressure applied to the insertion site for 20 min.

### 2.5. Toxicity monitoring

Patients were monitored for evidence of muscle toxicity via physical exam and serial CK measurements daily for 2 days. Systemic toxicity was monitored via daily WBC counts and EKG to monitor for QT prolongation. Defined follow-up for tumor response occurred at 0-, 2-, 6-, and 12-weeks post-infusion.

### 2.6. Tumor response

Response was defined by the RECIST 1.1 (https://recist.eortc.org/recist-1-1-2/): Complete Response (CR), Partial Response (PR), Stable Disease (SD), or Progressive Disease (PD) at 12 weeks post IA infusion.

### 2.7. Plasma and tissue collection/analyses

A peripheral venous blood sample was obtained prior to IA infusion and at time 0 (immediately before the end of 15 min infusion), 0.5 h, 1 h, 2 h, 4 h, 6 h, 8 h, 24 h, and 48 h after treatment.

Three, 6 mm Miltex^®^ punch biopsies (Integra LifeSciences Corp., Princeton, NJ), or five 18-gauge Bard Monopty^™^ core biopsies (C. R. Bard, Inc., Murray Hill, NJ), for deeper lesions of separate representative in-transit melanoma or sarcoma lesions were obtained prior to the IA infusion and at 24 h post-infusion. Punch biopsies were obtained in a similar manner for normal skin or tissue surrounding the tumor in the treatment field at the same time points for comparison.

CBL0137 quantitation in human K2EDTA plasma used the validated LC/MS/MS method. Blood samples were collected with K2EDTA and processed into plasma with CBL0137-[13C_4_] as an Internal Standard (IS). Aliquots were appropriately processed and then injected onto an LC-MS/MS system for analysis. For comparison of pharmacokinetics, data was referenced from a previous Phase I clinical trial of intravenous CBL0137 (NCT01905228) [16].

Tissue samples were combined with four volumes of 5 mL/mg collagenase in PBS pH 7.4 containing 0.5 mM calcium chloride and allowed to soak overnight at room temperature. After overnight incubation, all samples were thoroughly homogenized by sonication. Blank human skin tissue was homogenized by the same process and used as the matrix for preparation of quality control samples and calibration standards. Processed aliquots of supernatant were combined with 1 volume of water containing IS (0.2 μg/mL of CBL0137-[13C_4_]) and analyzed by a similar LC/MS/MS method used for plasma samples.

### 2.8. Statistical analyses

No formal statistical analyses were conducted. Data were presented descriptively, including a detailed patient listing with patient and tumor characteristics. Treatment-related adverse events were summarized in a table, categorizing the frequency and severity of events by system organ class (SOC) and preferred term (PT). Additionally, PK parameters were illustrated through graphical representations. All data are reported without statistical testing, focusing on descriptive summaries of the clinical observations.

## 3. Results

### 3.1. Patient data

From January 2020 through March 2023 five patients (3 sarcoma, 2 melanoma) were enrolled and treated in the rapid dose escalation protocol at Roswell Park Comprehensive Cancer Center. Patient demographics and tumor characteristics are summarized in Table 1 including prior treatment. The mean age of treated patients was 62.2 years with 3 males and 2 females. All patients had received some form of prior treatment with disease progression. The majority of patients (60 %) enrolled in this study would not have been candidates for either ILP or ILI as standard regional therapy approaches due to either a thrombosed major vein, severe contrast allergy that would preclude guiding a catheter across the major thoracic vessels, or proximity of tumor on the extremity that would prevent the placement of a tourniquet.

### 3.2. Treatment outcome and toxicity

The best noted response was stable disease in 1 patient with progressive disease in the remaining (Table 1). Despite being a Phase I study, the 1st patient treated continues to have stable disease >4 years from the infusion and maintained ambulatory status while avoiding the remaining option of above-knee amputation. Toxicity was minimal with 2 related grade 1 adverse events reported across the entire study and were due to discomfort due to catheter placement and tissue biopsy (Table 1). IA infusion of CBL0137 did not produce any changes in baseline CK levels, CBC, or EKG changes (data not shown).

### 3.3. Pharmacokinetics

**Fig. 3. –:**
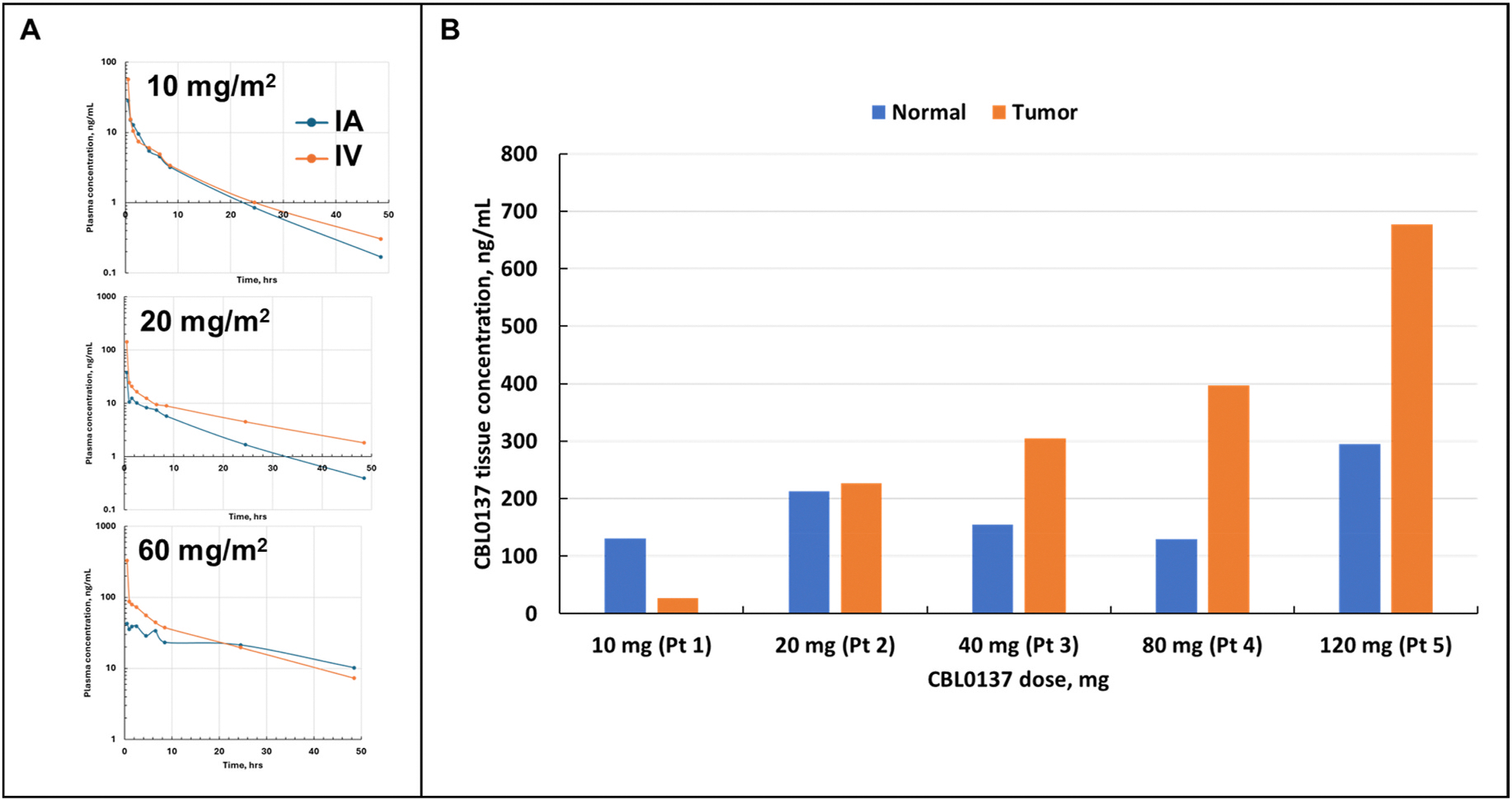
CBL0137 preferentially accumulates in tumor tissue after IA infusion. **A**) Comparison to PK values from similarly dosed IV patients, shows dose dependent lower initial concentrations for IA delivery suggesting increased drug retention in tissues. **B)** Doses above 40 mg of CBL0137 were associated with increased tumor uptake of CBL0137 compared to similarly exposed normal surrounding tissue at 24 h following IA infusion.

CBL0137 concentration in plasma shows a direct correlation to dose with a slower clearance noted at higher doses which was consistent based on absolute does or correcting for BSA (Fig. 1D). Correcting for BSA, the 20, 40, and 120 mg IA dose patients closely approximated 10, 20, and 60 mg/m^2^, respectively, and allowed for near direct comparisons to a prior intravenous study of CBL0137 over the same time points [16]. Comparing the IA to the intravenous route of similarly matched CBL0137 doses, the Cmax was on average 8.6-fold lower (range 5.6–13.4) in the IA infusion treated patients (Fig. 1E). Upon close inspection of the initial time points, comparisons of 10, 20, and 60 mg/m^2^ doses of CBL0137 either IA or intravenous showed an immediately lower systemic drug concentration for IA delivery (Fig. 3A). This substantial initial drop in plasma concentrations after IA administration as compared to intravenous delivery was more notable as the dose increased, suggesting immediate and increased retention in the targeted extremity that was dose-dependent. These results confirm that the IA route is associated with decreased systemic levels of CBL0137, well below the systemic MTD making isolation of the circulation or disposal of the venous effluent unnecessary.

### 3.4. CBL0137 tissue concentrations

Sampling was performed prior to, and 24 h after, IA infusion of both tumor and normal tissues of the treated extremity. Examining CBL0137 concentrations in tumor tissue and corresponding normal tissue demonstrated preferential uptake into tumor tissue (Fig. 3B). At doses ≥40 mg, there was an average 2 to 3-fold increase in CBL0137 concentration in tumor tissue as compared to normal tissue consistent with preclinical observations [15].

## 4. Discussion

The rapid dose escalation of IA CBL0137 proved feasible with minimal to no toxicity. Unlike melphalan-based regional therapies, the risk of tissue loss due to ischemia from tourniquet placement and circulatory isolation was eliminated. All infusions occurred in an interventional radiology suite, without the need for the OR or general anesthesia as is typically required with standard ILI or ILP. The infusion time of 15 min was much faster than the extensive operative set up and wash out time which significantly adds to the typical ILI or ILP drug circulation times of 30 and 60 min, respectively. Accepting the limitations of only having 1 patient per cohort for direct comparison of IA versus IV dosing, the rapid drop in plasma concentrations of CBL0137, the lower systemic Cmax, and preferential tumor accumulation with IA infusion suggests a favorable delivery route. Due to a lack of tumor tissue taken during the IV clinical trial, comparisons of tumor tissue CBL0137 uptake between IA and IV delivery methods were not possible. While not focused on response rates, this trial did demonstrate efficacy even at the low starting dose of 10 mg as disease stability was achieved in a patient who failed prior melphalan-based ILI and was able to remain ambulatory and avoid amputation. Furthermore, patients who would have been excluded as candidates for an ILI or ILP were able to be treated in this study. In fact, unlike ILI or ILP, the intra-arterial catheter could be placed retrograde with one patient having a radial artery catheterization to treat the ipsilateral extremity.

As IA infusion appeared to be well-tolerated with no vascular or tissue toxicity and as CBL0137 is tumor agnostic, this method may be extended to any site a catheter can be placed or directed. Technically demanding regional therapies such as isolated liver perfusion or isolated pelvic perfusion could be greatly simplified and become accessible to a greater number of patients [22,23]. CBL0137 has inherent properties that make it an attractive option for IA delivery including a quick tumor tissue uptake obviating the need for a tourniquet and unique downstream effects for potential synergism with other therapies. CBL0137 is capable of inducing Z-DNA formation, a left-handed twisting of standard DNA, within tumor cells and cancer-associated fibroblasts leading to an immunogenic cell death [24,25]. Combined with existing checkpoint inhibitor immunotherapy, IA infusion of CBL0137 would be hypothesized to elicit anti-tumor immune responses in immunotherapy refractory or resistant tumors as suggested preclinically [24,26] and will be a focus of future clinical studies. Furthermore, complex limb volume and correction calculations required for melphalan were omitted for fixed dosing of CBL0137 in this trial. As a biologically active agent that is fundamentally different than chemotherapy, a simplified dosing schema coupled with the ease of administration will likely facilitate combinatorial Phase II trials to examine efficacy. Future studies will examine molecular markers of CBL0137 downstream effects including levels of FACT subunits, p53 mutation influence on responsiveness, and induction of Z-DNA in tumor cells.

In summary, IA infusion of CBL0137 was well tolerated, feasible, and produced highly favorable PK and tumor/normal tissue concentration data analogous to preclinical studies. These early promising results lay the foundation for future trials and a full exploration of synergy with immunotherapy in the clinical setting.

## Figures and Tables

**Table 1:** Study details.

	CBL0137 (mg)	Age (years)	Sex	Weight (kg)	BSA	ECOG status	Tumor type	Stage	Grade	Location	Time from diagnosis (years)

Patient 1	10	55	M	99.2	2.29	1	leiomyosarcoma	II	intermediate	right popliteal area	3
Patient 2	20	57	F	69.9	1.77	2	dedifferentiated liposarcoma	II	low	left thigh	4
Patient 3	40	55	M	89.7	2.06	0	in-transit from acral lentiginous melanoma	IIIC	NA	left foot	12
Patient 4	80	62	F	131.8	2.38	0	in-transit from acral lentiginous melanoma	IIIB	NA	left thumb	4
Patient 5	120	82	M	70.1	1.84	2	UPS	II	high	left thigh	1

	Prior Chemotherapy	Prior Radiation Therapy	Prior Immunotherapy	Prior Regional Therapy	Prior Injectable Therapy
Patient 1	MAI adjuvant	50 Gy neoadjuvant	none	ILI melphalan + actinomycin D	none
Patient 2	MAI 4 cycles, eribulin + denosumab	50 Gy neoadjuvant, 20 Gy palliative	none	not possible - thrombosed femoral vein, proximity on thigh	none
Patient 3	dabrafenib-trametinib	NA	adjuvant interferon	none	none
Patient 4	none	NA	adjuvant ipilimumab	not possible - severe contrast allergy prevented placement of catheters	imiquimod/adpalene topical
Patient 5	none	50 Gy palliative	none	not possible - proximity on thigh	none

	IA Response	Alive	Adverse Event
Patient 1	SD	Y	NA
Patient 2	PD	N	NA
Patient 3	PD	Y	NA
Patient 4	PD	Y	NA
Patient 5	PD	N	Grade 1*

UPS – Undifferentiated Pleomorphic Sarcoma.

NA – Not Applicable.

MAI – Mesna, Doxorubicin, Ifosfamide.

Gy – Gray.

ILI – Isolated Limb Infusion.

SD – Stable Disease.

PD – Progressive Disease.

* pain/irritation at suture site.

## Data Availability

The data generated in this study are available upon request from the corresponding author.
